# Cell cycle exits and U-turns: Quiescence as multiple reversible forms of arrest

**DOI:** 10.12703/r/12-5

**Published:** 2023-03-08

**Authors:** Martha Sharisha Johnson, Jeanette Gowen Cook

**Affiliations:** 1 Department of Biochemistry and Biophysics, University of North Carolina at Chapel Hill, NC, USA

**Keywords:** Cell cycle, G_0_, proliferation, quiescence

## Abstract

Cell proliferation control is essential during development and for maintaining adult tissues. Loss of that control promotes not only oncogenesis when cells proliferate inappropriately but also developmental abnormalities or degeneration when cells fail to proliferate when and where needed. To ensure that cells are produced at the right place and time, an intricate balance of pro-proliferative and anti-proliferative signals impacts the probability that cells undergo cell cycle exit to quiescence, or G_0_ phase. This brief review describes recent advances in our understanding of how and when quiescence is initiated and maintained in mammalian cells. We highlight the growing appreciation for quiescence as a collection of context-dependent distinct states.

## Introduction

**Figure 1.**
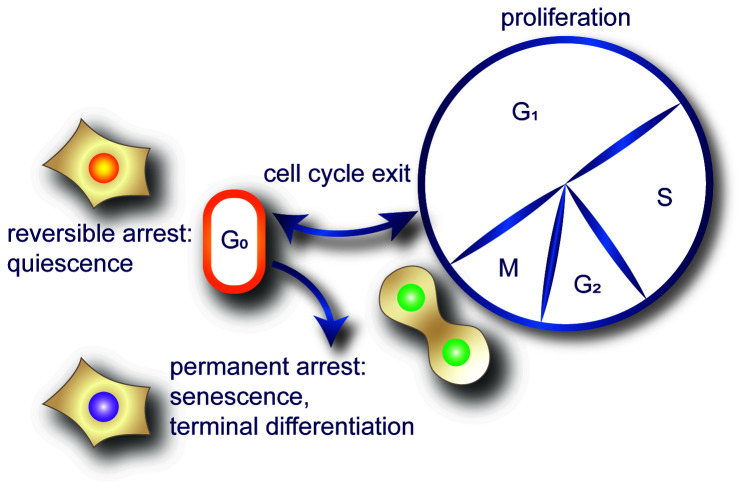
The cell division cycle consists of four proliferating phases—G_1_, S, G_2_, and M—and the reversible exit phase known as G_0_. The three main classifications of arrested cells are quiescence, senescence, and terminal differentiation. Quiescence is reversible, whereas senescence and terminal differentiation are generally permanent. Permanent arrest is assumed to be preceded by quiescence, as implied by the diagram, but is still not clear.

Proliferation is coordinated by a network of specific protein-DNA and protein-protein interactions that act in sequence to duplicate a cell. Proliferation is achieved by progression through the cell division cycle. This cycle includes two gap phases, G_1_ and G_2_, a DNA synthesis phase known as S phase, and M phase (mitosis), in which the replicated chromosomes are segregated, and cells divide (illustrated in Figure 1 and reviewed in 1–8). Transitions between phases are regulated by a series of checkpoint signaling mechanisms. When proliferation is no longer favored, cells should exit the cycle into an arrested state. Exit to an arrested state is normal and happens in response to a wide variety of external or internal stimuli. Controlled transitions between proliferation and arrest are crucial for normal development and tissue homeostasis to replace damaged cells, but controls must also prevent uncontrolled proliferation, which is a hallmark of cancer^9^. The cell cycle itself is reasonably well defined, but cell cycle exit is less well understood.

Common characteristics of arrested cells are the absence of both DNA synthesis (S phase) and cell division (M phase). Arrested cells continue to perform cellular and metabolic functions, transcription, and translation. Arrested cells exist in one of three categories of non-dividing states: terminal differentiation, senescence, and quiescence. These arrests can be permanent (differentiation and senescence) or transient for brief or very prolonged periods (quiescence). Arrested states are distinguished from one another by their unique features and degree of reversibility (reviewed in 6,10,11).

Terminal differentiation is linked with generally irreversible exit from the cell cycle. This non-dividing state represents a common cellular state in many adult organisms. Once cells terminally differentiate, they become non-responsive to all proliferative signals^12^. Senescence, another form of irreversible exit, is a reaction to cellular stress, oncogene activation, and DNA damage, including telomere erosion^13^. On the other hand, quiescence, unlike the irreversible arrests of senescence and terminal differentiation, is a reversible state and is often referred to as G_0_ phase.

Examples of signals that can induce quiescence are reduced growth factors or nutrients^14^, high cell density^15^, or various cellular stresses^16,17^. Cells can later leave quiescence, re-enter the cell division cycle, and divide. The ability to re-enter the cell cycle is vital to homeostasis and repair in tissues. Quiescence is a characteristic of a wide range of diverse cell types such as organ- and tissue-specific adult stem cells, including skin, muscle, and neural stem cells, as well as fibroblasts and lymphocytes^6,10,11^. Quiescence is also a mechanism for cancer cells to avoid common therapies that target DNA synthesis or mitosis^18–20^. The prevalence and importance of quiescence in biological systems make it imperative that we understand the molecular and cellular basis of quiescence. There are excellent reviews of cell cycle control available^1–8^; in this concise review, we explore recent developments in understanding cell cycle exit to quiescence (G_0_). For brevity, we focus on quiescence in mammals, but many of the mechanisms described are evolutionarily conserved in other organisms.

## Characteristics of quiescent cells

Cell cycle arrest is accomplished by repressing the cell cycle machinery that drives active proliferation. The cell cycle is a unidirectional series of phases driven by cyclin-dependent kinases (CDKs) and their activating subunits, the cyclins. Cyclin-CDK complexes control the cell cycle by phosphorylating proteins that carry out the major activities in each phase, such as DNA replication in S phase or chromosome segregation in mitosis. There are multiple CDKs and cyclins, and different CDK complexes govern different cell cycle phases (reviewed in 4,21). The activities of CDKs that normally control cell cycle progression are all low in quiescent cells. The drop in CDK activity can be the result of reduced cyclin expression or increased expression of dedicated protein CDK inhibitors (CKIs) or both^22^. Moreover, the vast majority of additional genes and activities that accomplish DNA synthesis and mitosis are repressed in quiescent cells^23,24^.

Although quiescent cells can remain arrested for long periods of time, they maintain their viability and their ability to proliferate when stimulated. To maintain viability, quiescent cells protect themselves from accumulating damage, such as damage from reactive oxygen species. For example, detoxifying enzymes such as superoxide dismutase and glutathione peroxidase are induced in quiescent cells^25,26^. Quiescent cells are also characterized by a substantial decrease in oxidative phosphorylation in favor of glycolysis, reduction in energy production (lower ATP concentrations), and less overall biosynthesis^27–30^.

In general, quiescent cells express low levels of proteins required for cell cycle progression. Several cell cycle-associated biomarkers, such as Ki67 and PCNA, two proteins that are uniquely expressed in proliferating cells but not quiescent (or senescent or terminally differentiated) cells, are commonly used to analyze clinical samples such as cancer biopsies^31^. Recent studies have identified additional molecular markers of quiescence, some of which are induced rather than repressed in G_0_. In the following section and Table 1, we describe some of these unique molecular features of quiescent cells.

**Table 1.** Molecular markers of quiescence that are highlighted in this review.

Changed in quiescence	Molecular marker
Cyclins	Reduced cyclin expression
CDK inhibitors	Increased expression of p21 and p27
Cyclin-dependent kinase (CDK) activity	Low CDK activity
Unlicensed DNA replication origins	Mini-chromosome maintenance (MCM) complexes are not loaded
Retinoblastoma protein (RB)	Low RB phosphorylation
DREAM transcriptional repressor complex	DREAM complex assembled
Ubiquitin E3 ligase anaphase-promoting complex	Substrates degraded (e.g., Skp2)
Protein translation	Reduced translation (low phospho-S6)
Autophagy and lysosomal function	Increased lysosomal gene expression
Ciliogenesis	Primary cilium formed

### Distinct molecular markers of quiescence

One challenge in the field is distinguishing quiescent cells from G_1_ cells. Most commonly used cell cycle assays, such as immunostaining or flow cytometry, cannot differentiate G_0_ cells from early G_1_ cells. Both G_0_ and G_1_ cells contain the same DNA content and have similar CDK activity, size, and morphology. Is G_0_ *qualitatively* different from a very long G_1_ phase? Coller et al. defined unique transcriptional profiles of G_1_ fibroblasts compared with G_0_ fibroblasts^23^. This observation was corroborated by proteome profiling of growth factor-depleted cells compared with G_1_ cells; nearly half of the proteins that changed between G_0_ and G_1_ are not cell cycle regulated during normal proliferation^27^. More recently, Min and Spencer analyzed transcriptional profiles of epithelial cells and also found distinct gene expression changes in G_0_ cells compared with G_1_ cells^32^. Taken together, the unique patterns of gene and protein expression demonstrate that G_0_ is not simply a long G_1_ but is a separate biological state. In this section, we describe several of the most commonly studied markers of quiescent cells.

### Low CDK activity

Sequential waves of distinct CDK activities control cell cycle progression. G_1_ phase CDKs are cyclin D/CDK4, the highly related cyclin D/CDK6, and cyclin E/CDK2 complexes. Cyclin D expression and accumulation are induced by extracellular signals such as mitogenic growth factors^33^. Cyclin D-associated kinases indirectly stimulate the transcription of genes encoding the cyclins that function in the subsequent cell cycle phases^5,34,35^. When cells exit the cell cycle to G_0_, cyclin D expression is suppressed, leading to the loss of the other cyclins as well. In addition, a family of CDK inhibitor proteins that bind and inactivate CDK enzymatic activities are typically induced in arrested cells. These Cip/Kip family CDK inhibitors (CKIs) are p21^Cip1^ and p27^Kip1^, which are broadly expressed, and CKI p57^Kip2^, whose expression is largely restricted to specific differentiated cell types in adults^22,36–38^. The combination of cyclin down-regulation and CKI accumulation suppresses CDK activity in quiescent cells.

### Unlicensed DNA replication origins

DNA replication in S phase requires pre-loading chromosomes with DNA helicase components, the mini-chromosome maintenance complex (MCM), during G_1_ phase. The process of MCM loading is termed “replication origin licensing.” During proliferative cell cycles, MCM loading is active during G_1_ phase but is blocked starting from the G_1_/S transition until anaphase to avoid re-licensing and re-replicating any genomic loci (review in 39–41). CDK activities in S, G_2_, and M phases contribute to inhibiting unscheduled MCM loading by inactivating several essential MCM loading factors: Cdt1, Cdc6, and ORC^42,43^. Interestingly, although CDK activity is very low in quiescence, mammalian G_0_ cells do not load MCM complexes, and replication origins remain unlicensed^44,45^. CDK-independent mechanisms that prevent MCM loading include low Cdt1 and Cdc6 expression plus proteolysis of the Cdc6 loading factor by ubiquitin-mediated degradation^46,47^. However, a stable Cdc6 variant co-expressed with ectopic Cdt1 could support MCM loading in G_0_ cells only when both were significantly overexpressed, suggesting that other as-yet-unidentified, MCM loading inhibition mechanisms prevent inappropriate replication origin licensing in quiescent cells^46^.

### Low/absent retinoblastoma protein phosphorylation

The retinoblastoma protein (RB) is a negative regulator of proliferation. RB binds the E2F class of transcription factors to repress E2F target genes; E2F-regulated genes include many whose expression is necessary for S phase initiation and progression, including other cyclins^33,48^. In proliferating cells, CDK-mediated RB hyper-phosphorylation in late G_1_ relieves this transcriptional repression, whereas in early and mid-G_1_, RB mono-phosphorylation fine-tunes G_1_ gene expression and chromatin organization in ways that are not yet fully elucidated^49–51^. The CDKs that inactivate RB in G_1_ phase are typically cyclin D/CDK4, cyclin D/CDK6, and cyclin E/CDK2 complexes^4,8,52^, and a recent report suggests that they may work together to maintain RB phosphorylation and allow S phase initiation^53^. This collaboration between CDK4 (or CDK6) and CDK2 contrasts with previous models of sequential RB phosphorylation, first by cyclin D-associated CDK and then by cyclin E-associated CDK activity. Because all the major CDK activities are low in quiescent cells, RB in its repressive hypo-phosphorylated or un-phosphorylated form in G_0_, and RB is required to maintain quiescence fully^54^.

The development of new tools for live single-cell analysis has launched recent updates to our understanding of cell cycle exit and re-entry. The conversion of RB from hypo-phosphorylated to hyper-phosphorylated in late G_1_ phase has been extensively studied in cells released from quiescence into G_1_ phase. It was assumed that these findings also applied to cells progressing through G_1_ from mitosis and that RB hyper-phosphorylation was primarily a late G_1_ event. What is now emerging is a greater appreciation that the first G_1_ phase after quiescence is not typical of the G_1_ phases after mitosis in actively proliferating cells^55,56^. Strikingly and unlike early G_1_ during cell cycle re-entry, RB is already hyper-phosphorylated in most very early G_1_ cells after mitosis. In many analyzed cell populations, a significant but variable subpopulation of spontaneously quiescent cells enters G_1_ from mitosis with hypo-phosphorylated RB^32,57–59^. Instead of RB hypo-phosphorylation being the typical state in all early G_1_ cells as previously presumed, hypo-phosphorylated RB may, in fact, be restricted to quiescence. This distinction could not have been made by studying cell populations using ensemble molecular assays such as immunoblotting.

### DREAM complex assembly

RB is a member of the family of pocket proteins that includes two other members: p130 and p107. In cells that have fully exited to quiescence, repression of genes encoding proteins that promote cell cycle progression is the responsibility of the p130 protein more so than RB or p107^60^. To maintain repression, p130 accumulates and assembles with the MuvB protein complex (consisting of LIN9, LIN54, LIN37, LIN52, and RBBP4 proteins) and with a transcriptionally repressive E2F family member, E2F4. This assembly is termed the DREAM complex, and it represses most cell cycle gene expression during quiescence^61,62^. DREAM disruption alone can induce cell cycle re-entry^63^. The complex does not assemble in G_1_ phase during normal proliferation and is thus a unique characteristic of quiescent cells. In fact, DREAM must be disassembled by CDK-mediated phosphorylation during cell cycle re-entry^64^. DREAM assembly in quiescence is dependent on unique phosphorylation of the LIN52 subunit to connect the five-member MuvB complex to p130 and E2F4. This phosphorylation is carried out by a kinase, DYRK1A, whose activity toward LIN52 is induced in G_0_. What controls DYRK1A kinase activity toward LIN52? The upstream regulators of DYRK1A are still unclear, although the LATS1 and LATS2 kinases of the Hippo signaling pathway are candidates^65^.

### Anaphase-promoting complex

Cyclin down-regulation in quiescent cells is due to a combination of low cyclin gene expression and active ubiquitin-mediated degradation by the ubiquitin E3 ligase, the anaphase-promoting complex/cyclosome (APC/C)^66^. In proliferating cells, APC/C directly targets mitotic cyclins for degradation, beginning in anaphase and continuing through the end of G_1_ phase^67^. APC/C is also active during G_0_ and is required to maintain quiescence^68^. APC/C indirectly inhibits CDK activity by inactivating a negative regulator of two CKIs: p21^Cip1^ and p27^Kip1^^69^. This negative regulator, S phase kinase-associated protein 2 (Skp2), is a cullin-type E3 ligase subunit that stimulates p21 and p27 degradation^70,71^ and is reviewed in 72,73; thus, APC/C-mediated Skp2 degradation promotes CKI accumulation and therefore CDK inhibition. Interestingly,****** RB may play a direct role in this APC/C-mediated CKI regulation independently of its role as a transcriptional repressor of E2F-target genes by physically recruiting Skp2 to APC/C for promoting Skp2 degradation^74,75^.

### Protein translation

A specific metabolic change during quiescence is a general decrease in the rate of translation. The signaling pathway that primarily controls protein synthesis in response to growth factors and nutrients is the mammalian target of the rapamycin (mTOR) signaling pathway, and one of its downstream targets is ribosomal protein S6. Nutrient or growth factor depletion inhibits mTOR signaling. Thus, low pS6 phosphorylation is an indicator of reduced protein synthesis, another unique feature of quiescence compared with proliferation^76,77^. Because quiescent cells do not typically grow in size, and protein synthesis is a substantial energy-consuming process, quiescent cells conserve energy by reducing protein production^78^. This change contrasts with senescent cells that have relatively higher rates of active protein synthesis^79^.

### Autophagy and lysosomal function

Autophagy is a process in which cellular components, like unneeded proteins and damaged organelles (i.e., damaged mitochondria), are engulfed by autophagosomes and delivered to lysosomes for degradation and recycling of their components. This process of autophagy maintains cellular homeostasis^80^ and regulates cell growth^81,82^. Quiescent cells depend on autophagic processes for preserving cellular viability during arrest^83^. In quiescent stem cells, both autophagosomes and autolysosomes form in response to growth factor removal^84–86^. Cells that have been quiescent from growth factor depletion for substantial lengths of time display reduced autophagy activity. To counteract this down-regulation, they induce lysosomal gene expression and function^87,88^; the cause of reduced autophagy is still unclear. This induced lysosomal function is required to preserve the ability to efficiently re-enter the cell cycle^87^. These findings suggest that lysosome function sets the depth of quiescence and prevents an inappropriate descent into senescence.

### Ciliogenesis

Quiescent cells also undergo physical changes in addition to biochemical changes. Most quiescent cells assemble a microtubule-based projection known as the primary cilium at the plasma membrane. This primary cilium acts as a sensory structure (like an antenna) that is key to cell growth and differentiation^89^. During the establishment of quiescence, the single centrosome inherited from the previous mitosis migrates to the apical cell surface to form the base for the primary cilium. Exit from quiescence back into an active proliferating cycle is accompanied by cilium resorption^90,91^. The precise timing and relationship between ciliogenesis and other cell cycle exit markers are poorly understood. The presence of a primary cilium may not only be a marker of quiescence but also may contribute to maintaining arrest by suppressing proliferative signal transduction pathways^92^.

## The process of cell cycle exit

**Figure 2.**
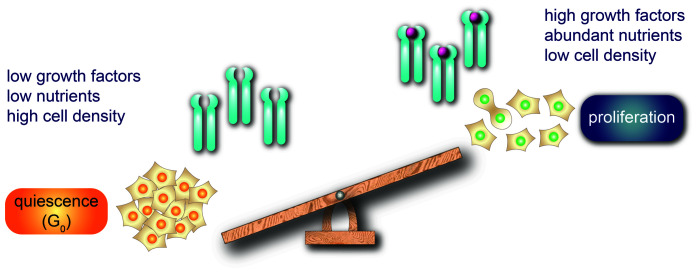
The proliferation-quiescence decision determines whether a cell exits the cell cycle or commits to another cycle. The balance of pro-proliferative and anti-proliferative conditions—such as cell density, the concentration of mitogenic growth factors, and available nutrients—controls that decision. Precisely when the decision is made is still unclear. The turquoise symbols represent signaling receptors, and the magenta spheres represent growth factors.

The commitment to exit the cell cycle to a quiescent state is sometimes referred to as the “proliferation-quiescence decision.” Multiple signals are integrated to influence the decision, including internal signals such as cellular stress and DNA damage and external signals such as growth factor signaling and cell-cell contacts. Figure 2 illustrates the concept of a balance between pro-proliferative signals, such as abundant growth factors and low cell density, and pro-quiescence signals, such as reduced growth factors or nutrients or high cell density. When is the decision made relative to the final mitosis, and exactly how is the decision implemented? Answers to these questions are being actively pursued.

### Decision timing

**Figure 3.**
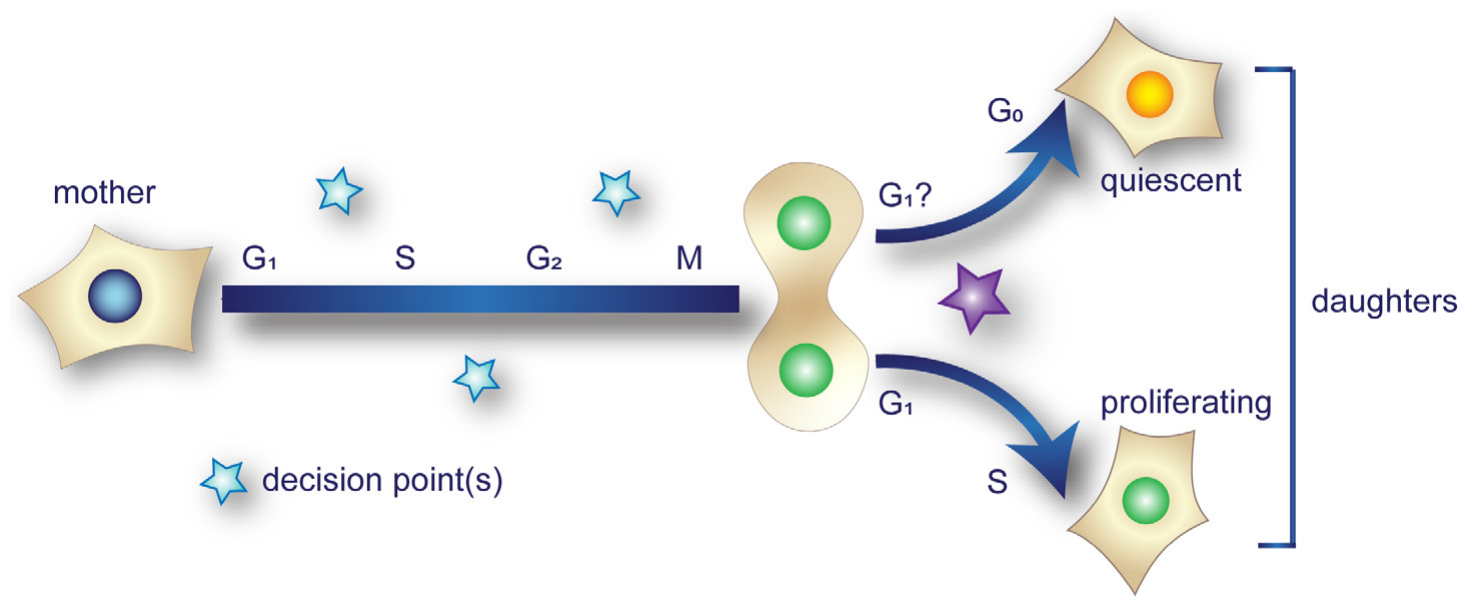
Timing of the proliferation-quiescence decision has long been thought to occur during G_1_ of daughter cells (purple star). Recent studies, however, indicate that the decision to exit the cell cycle can also be made during the mother’s cell cycle (cyan stars).

For many years, the proliferation-quiescence decision was presumed to be an exclusively G_1_ event^14,93^. Recent developments have brought this assumption into question by tracking cells through their final cell cycle and into the early stages of quiescence, as illustrated in Figure 3. Several studies have shown correlations between signaling or stresses in the mother cell prior to the final division and the fate of the resulting daughter cells. Increased stress or decreased growth factor signaling in the mother increases the likelihood that daughter cells become quiescent even if the daughters are “born” into conditions optimal for proliferation. For example, replication stress in the mother cell increases the levels of the CDK inhibitor p21^Cip1^ such that daughters are born with particularly high p21^Cip1^ and, thus, low CDK activity^59,94–96^. High cell density in mothers suppresses cyclin D expression relative to the p27^Kip1^ CDK inhibitor and increases the likelihood of quiescent daughters^97^. Similarly, cellular stress signaling in the mother increases the probability of spontaneously quiescent daughters by suppressing cyclin D expression^32^. Moreover, brief periods of low growth factor exposure or interrupted growth factor signaling in the mother cell increase the likelihood of quiescent daughters^98^, and this effect seems irrespective of the mother cell cycle phase in which growth factors were reduced^99^. These recent studies indicate that the proliferation-quiescence decision can be made during the mother’s cell cycle prior to the final mitosis. Moreover, stochastic differences in stresses experienced by mother cells underlie some of the intercellular variability in proliferation that is widely observed in genetically identical cells grown in identical conditions^100^. It is not yet clear whether proliferation-quiescence decision timing is similar in most cell types or in response to most inducers of quiescence.

### Transitions to G_0_ from different cell cycle phases

Based on observations that quiescent cells usually have G_1_ DNA content, cells exit the cycle to quiescence most commonly from the G_1_ phase. However, cells can leave the cycle from the G_2_ phase after duplicating their DNA but before mitosis. These arrested cells have a G_2_ DNA content but express molecular markers of G_1_ or G_0_, or both. Skipping mitosis is a normal developmental step for some cell types that are destined to be polyploid^101^, but it can also happen in response to cellular stress. Exit from G_2_ is most readily observed in response to replication inhibitors during S phase, suggesting that replication stress or small amounts of unreplicated DNA can block mitotic entry^102,103^. These arrested cells frequently enter permanent arrest as senescent cells^104^. It is not known whether they transit through a typical quiescence molecular state on the way from G_2_ to senescence or whether they bypass quiescence altogether.

### Quiescence heterogeneity

Appreciation has grown in recent years that quiescence is not uniform but rather is a heterogeneous collection of related but non-identical states. Molecular signatures in G_0_ cells can vary based on cell type and the inducer of quiescence. For example, fibroblasts induced into quiescence by growth factor deprivation, contact inhibition, or loss of adhesion displayed gene expression profiles that were remarkably different from one another, although some changes were common^23^. Similarly, quiescent epithelial cells show expression profiles that correlate with the method of quiescence induction^32,105^. There are also cell type-specific differences that contribute to heterogeneity. For example, Wnt signaling, which is associated with developmental proliferation control^106^, was active in quiescent myoblasts but not in quiescent fibroblasts^107^.

Even within a single-cell type and common quiescence inducer, cells can vary in other aspects of quiescence. In particular, cells that have been in G_0_ for long periods of time have additional protein and gene expression features compared with cells that exited the cell cycle more recently^87^. Such cells can be described as “deeply” quiescent because they require higher proliferative signals and more time after stimulation to re-enter the cell cycle. They may also develop additional features during long arrest, such as highly condensed chromosomes that are not characteristic of early G_0_ cells^108^. A proteome profile of serum-deprived cells also showed evidence for global chromatin-related changes in addition to the expected down-regulation of proliferation markers^27^. Such cells must overcome higher barriers than similar cells in a “shallower” quiescent state to return to proliferation^109,110^. Length of arrest is likely not the only determinant of deep or shallow quiescence. Cell type and environmental cues can also influence quiescence depth. For example, muscle stem cells ascend from their resting deep quiescent state to a shallower state termed by the authors “G alert” in response to injury at distant sites. Specific signals stimulate these muscle cells to either prepare to enter S phase more readily or return to deeper quiescence^111^.

### Signaling changes that induce quiescence

Changes in the activity of signaling pathways that affect core cell cycle machinery, such as CDKs, can induce cell cycle exit to quiescence; we highlight several examples in this subsection. Transcription of the *cyclin D* gene is stimulated by a variety of common growth factor signaling pathways, including those that include the ras GTPase and a cascade of kinases culminating in ERK/MAP kinase activation^33^. Reduced signaling through these pathways reduces cyclin D expression. Alternatively, signaling that increases expression of the CDK inhibitors can tip the balance toward quiescence, such as induction of the p27^Kip1^ CKI by transforming growth factor beta (TGF-β) signaling^112^. In contrast, some pathways can either activate or repress quiescence based on the cell type and conditions. For example, the Notch pathway can activate proliferation by inducing cyclin D expression^113,114^ or induce quiescence by interfering with cyclin D/CDK4 activity^115^ depending on cell type and conditions^114,116^.

Commonly used drugs can also target signaling pathways that are responsible for proliferation. Metformin, a drug used to treat diabetes and some cancers^117^, induces both autophagy and arrest in G_0_/G_1_ phase. This arrest is mediated by altered gluconeogenesis which activates adenosine monophosphate-activated protein kinase (AMPK), which then represses the mammalian target of the rapamycin (mTOR) pathway^118,119^. As a result, protein translation is inhibited, and cells exit to quiescence.

The mTOR pathway is inhibited by rapamycin, a natural product. Other natural products are also being used to induce a G_0_ state. Some of these natural products target and inhibit specific signaling pathways that are active during proliferation. An additional example of these natural products is caffeine, a xanthine alkaloid that can have anti-cancer effects^120,121^. Increasing concentrations of caffeine inhibited cell proliferation and induced molecular markers of quiescence, such as RB hypo-phosphorylation^120,122,123^. The mechanism by which caffeine arrests cell proliferation includes cyclin D repression by activating the protein kinase A signaling pathway which ultimately activates the kinase GSK3β. Because active GSK3β may phosphorylate cyclin D to promote its degradation^124^, caffeine-induced GSK3β activation may contribute to cyclin D down-regulation which prevents G_1_ progression.

Environmental hazards and metal exposures also frequently inhibit proliferation, possibly by triggering quiescence^125,126^. Liu et al. observed that hepatocytes in mice exposed to copper (Cu), a toxic metal, underwent cell cycle arrest^127^. These cells down-regulated pro-proliferation proteins and mRNAs (such as the proliferative signaling kinase AKT), and they up-regulated anti-proliferative factors such as p21^Cip1^ and p27^Kip1^^127^. These metal-exposed cells also down-regulated the protein and mRNA levels of cyclin D, cyclin E, CDK2, and CDK4, all important proteins for progression through G_1_ into S phase. The authors concluded that copper metal exposure suppresses at least one of the pathways that transmit growth factor signals. Like many toxic metals, copper induces oxidative stress^128^, and excess oxidative stress damages DNA, lipids, and proteins and also inhibits proliferative signaling^129^. Suppressed signaling reduces CDK2, CDK4, cyclin E, and cyclin D levels which, in turn, promotes cell cycle exit.

The drug Dioscin, which is obtained from a natural steroid saponin, inhibits cancer cell proliferation by blocking the cell cycle through up-regulating CKIs and down-regulating cyclins and CDKs^130^. One of the targets inhibited by Dioscin is Skp2, a negative regulator of p21^Cip1^ and p27^Kip1 ^CKIs^131^. In contrast to indirect CDK inhibition induced by compounds such as Dioscin, cancer therapies directly targeting CDKs have also been developed. Drugs that specifically bind and inhibit CDK4 and CDK6 activities have shown promise in treating a subset of breast cancers^132,133^. Interestingly, prolonged CDK4 and CDK6 inhibition is only partially reversible^134^, and long-term drug treatment can induce senescence in cultured cells^135^. More recently, a class of drugs that inhibit CDK2, CDK4, and CDK6 simultaneously was reported^136^.

## Conclusions and future directions

A recent focus on single-cell assays and unbiased global transcriptome and proteome profiling has begun to reveal the true complexity of cell cycle quiescence. Quiescent cells have characteristic phenotypes and molecular markers that distinguish them from cells arrested in one of the proliferative cell cycle phases. Quiescent cells are also distinct from other kinds of arrests, such as cells responding to DNA damage checkpoint activation, although some features, such as low CDK activity, are similar^137,138^. Recent studies raise several questions that will be important to address in future investigations: (1) What are the molecular changes that occur during the process of cell cycle exit—which changes are early and which occur later? The process of cell cycle exit itself has not yet been well investigated, in part because the timing of cell cycle exit is highly heterogeneous among individual cells. (2) Do cells that are destined for permanent arrest pass through quiescence first, or do they follow a unique pathway? (3) What characterizes quiescence of different depths, and is progression from shallow to deeper quiescence gradual or stepwise? Analysis of the proliferation marker, Ki67, in cells undergoing spontaneous arrest or arresting from growth factor signaling inhibitors indicated a gradual loss of this particular marker for those specific arrests^139^, but is that behavior typical? (4) Similarly, do deeply quiescent cells transition to permanent arrest (senescence)? If so, how? (5) What features of quiescence are universal, and which features vary with cell type and arrest conditions? A validated set of molecular markers of quiescence could be useful for characterizing cells in patient biopsies. (6) Which molecular features of quiescent cells are integral to establishing and maintaining quiescence, and are some of these features primarily downstream effects rather than causes? A full understanding of how cells move into and out of different types of quiescence could be used to deliberately shift shallow quiescent cancer cells into permanent arrest or to induce stubbornly quiescent cells to proliferate in damaged or aged tissue. Further progress in defining quiescence will have implications for understanding developmental biology, aging, regeneration, and cancer biology.
